# Supplementary material for: Multi‐dataset identification of innovative feature genes and molecular mechanisms in keratoconus
Source: J Cell Mol Med. 2024 Sep 19;28(18):e70079. doi: 10.1111/jcmm.70079 (PMC11412914; doi:10.1111/jcmm.70079)
Supplement: Supplementary file 2 — Material S2. [file JCMM-28-e70079-s002.zip › 7.GSEA analysis/5.RNF217-AS1/KEGG_BASE_EXCISION_REPAIR.html]

Details for gene set KEGG\_BASE\_EXCISION\_REPAIR[GSEA]

|  || Dataset | RNF217-AS1.group.cls#h\_versus\_l |
| Phenotype | group.cls#h\_versus\_l |
| Upregulated in class | l |
| GeneSet | KEGG\_BASE\_EXCISION\_REPAIR |
| Enrichment Score (ES) | -0.50928664 |
| Normalized Enrichment Score (NES) | -1.5941187 |
| Nominal p-value | 0.030487806 |
| FDR q-value | 0.35485736 |
| FWER p-Value | 0.609 |
Table: GSEA Results Summary

  

Fig 1: Enrichment plot: KEGG\_BASE\_EXCISION\_REPAIR      
 Profile of the Running ES Score & Positions of GeneSet Members on the Rank Ordered List

  

| SYMBOL | TITLE | RANK IN GENE LIST | RANK METRIC SCORE | RUNNING ES | CORE ENRICHMENT || 1 | POLE | na | 10398 | 0.080 | -0.2678 | No |
| 2 | HMGB1 | na | 13739 | 0.033 | -0.3506 | No |
| 3 | NEIL1 | na | 13942 | 0.031 | -0.3439 | No |
| 4 | POLD4 | na | 14819 | 0.021 | -0.3607 | No |
| 5 | MUTYH | na | 15701 | 0.012 | -0.3814 | No |
| 6 | POLD1 | na | 16368 | 0.005 | -0.3985 | No |
| 7 | POLL | na | 16815 | 0.000 | -0.4112 | No |
| 8 | UNG | na | 17712 | -0.008 | -0.4338 | No |
| 9 | PARP4 | na | 17964 | -0.010 | -0.4368 | No |
| 10 | POLE4 | na | 18097 | -0.012 | -0.4360 | No |
| 11 | APEX1 | na | 18363 | -0.014 | -0.4380 | No |
| 12 | PARP2 | na | 19907 | -0.029 | -0.4708 | No |
| 13 | XRCC1 | na | 21243 | -0.043 | -0.4922 | Yes |
| 14 | POLD3 | na | 21754 | -0.048 | -0.4877 | Yes |
| 15 | SMUG1 | na | 22334 | -0.054 | -0.4826 | Yes |
| 16 | POLE3 | na | 22462 | -0.056 | -0.4640 | Yes |
| 17 | MPG | na | 22638 | -0.058 | -0.4459 | Yes |
| 18 | TDG | na | 22898 | -0.061 | -0.4290 | Yes |
| 19 | POLD2 | na | 23076 | -0.063 | -0.4090 | Yes |
| 20 | NEIL2 | na | 23209 | -0.064 | -0.3871 | Yes |
| 21 | NEIL3 | na | 23213 | -0.064 | -0.3614 | Yes |
| 22 | APEX2 | na | 23604 | -0.070 | -0.3447 | Yes |
| 23 | POLB | na | 24510 | -0.083 | -0.3375 | Yes |
| 24 | PCNA | na | 24885 | -0.089 | -0.3128 | Yes |
| 25 | LIG1 | na | 24995 | -0.090 | -0.2797 | Yes |
| 26 | OGG1 | na | 25132 | -0.092 | -0.2466 | Yes |
| 27 | LIG3 | na | 25250 | -0.094 | -0.2121 | Yes |
| 28 | MBD4 | na | 26501 | -0.116 | -0.2015 | Yes |
| 29 | PARP1 | na | 27719 | -0.142 | -0.1797 | Yes |
| 30 | PARP3 | na | 28283 | -0.156 | -0.1335 | Yes |
| 31 | NTHL1 | na | 29021 | -0.177 | -0.0839 | Yes |
| 32 | FEN1 | na | 31474 | -0.262 | -0.0494 | Yes |
| 33 | POLE2 | na | 32955 | -0.357 | 0.0511 | Yes |
Table: GSEA details [plain text format]

  

Fig 2: KEGG\_BASE\_EXCISION\_REPAIR      
 Blue-Pink O' Gram in the Space of the Analyzed GeneSet

  

Fig 3: KEGG\_BASE\_EXCISION\_REPAIR: Random ES distribution      
 Gene set null distribution of ES for **KEGG\_BASE\_EXCISION\_REPAIR**
